# Breast Tumor Ultrasound Image Segmentation Method Based on Improved Residual U-Net Network

**DOI:** 10.1155/2022/3905998

**Published:** 2022-06-25

**Authors:** Tianyu Zhao, Hang Dai

**Affiliations:** ^1^Medical Technology Department, Qiqihar Medical University, Qiqihar, Heilongjiang 161006, China; ^2^Foreign Language Department, Qiqihar Medical University, Qiqihar, Heilongjiang 161006, China

## Abstract

In order to achieve efficient and accurate breast tumor recognition and diagnosis, this paper proposes a breast tumor ultrasound image segmentation method based on U-Net framework, combined with residual block and attention mechanism. In this method, the residual block is introduced into U-Net network for improvement to avoid the degradation of model performance caused by the gradient disappearance and reduce the training difficulty of deep network. At the same time, considering the features of spatial and channel attention, a fusion attention mechanism is proposed to be introduced into the image analysis model to improve the ability to obtain the feature information of ultrasound images and realize the accurate recognition and extraction of breast tumors. The experimental results show that the Dice index value of the proposed method can reach 0.921, which shows excellent image segmentation performance.

## 1. Introduction

As a malignant disease with high mortality, breast cancer has seriously threatened the physical and mental health and life safety of women worldwide [1, 2]. According to the American Cancer Society, in 2017, 255180 new cases of breast cancer and 41070 deaths of breast cancer were reported in the United States. In China, there are about 66 thousand female breast cancer deaths each year, accounting for 7.82% of the total number of deaths from female malignant tumors. After breast cancer cells proliferate, free diffusion can occur. Breast cancer forms a tumor metastasis site through local invasion, intravascular infiltration, circulatory system, and/or lymphatic system transmission [3–5]. Clinically, the process of multiple organ disease caused by metastasis is one of the important factors leading to high mortality of breast cancer. The prognosis of patients with metastatic breast cancer usually is not good, and the average 5-year survival rate is only 27%. In the formation of clinically detectable cancer cell metastasis, often the patient's condition has developed into advanced tumor [6]. Thus, early detection, early diagnosis, and early treatment of breast cancer are of great significance.

With the development and application of computer technology, researchers choose to continuously train the tumor ultrasound image dataset to learn with the help of deep learning network and independently extract the information features in the dataset, so as to realize noninvasive tumor medical auxiliary judgment [7]. However, it should also be noted that, due to the deep structure of the depth network model, the gradient is easy to disappear when training the dataset to learn, which makes it difficult for the image segmentation network model to realize accurate and effective tumor region recognition.

In this study, U-Net network and attention mechanism module are combined to construct breast tumor ultrasound image segmentation model, improve image recognition accuracy, and provide auxiliary reference for medical staff. The contributions of the proposed method lie in the following:

1. The residual block is used to optimize and improve the U-Net network, and the residual mapping is obtained by convoluting the input features through the convolution layer, which can effectively solve the gradient disappearance problem of the deep network.
2. The hybrid attention mechanism module is added to the image segmentation model to optimize the training and learning process of the image segmentation model, enhance the information feature acquisition ability of the model, and support the accurate and efficient recognition of the image segmentation model.

The remaining chapters are arranged as follows. The second chapter introduces the relevant work in this field. The third chapter introduces the breast tumor ultrasound image segmentation method based on improved residual U-Net network.  In Chapter 4, experiments are designed to verify the performance of the proposed model. The fifth chapter is the conclusion.

## 2. Related Researches

Image segmentation is a technology to extract the region of interest in the image according to the pixel features (gray, texture, etc.). For breast ultrasound images, the region of interest is the breast nodule. It is necessary to extract the diseased nodule from the normal tissue region to provide input data for the next classification operation. At present, the main algorithms proposed by domestic and foreign scholars in the research of ultrasound image segmentation are threshold and edge method, region method, graph theory and clustering method, energy functional method, and neural network method [8].

Traditional image segmentation algorithms, such as threshold method, edge method, and region method, do not easily obtain ideal segmentation results directly. General researchers will make a lot of improvements and integration on the above algorithms. Liu et al. [9] proposed a framework for fully automatic segmentation of breast ultrasound image lesion area, which used Otsu-based adaptive thresholding (OBAT) method and morphological filtering method to locate the region of interest (ROI) and initialize the nodule contour [9]. Jiang et al. [10] used AdaBoost + Haar framework to locate a set of potential lesion locations in breast ultrasound images [10]. However, if only these methods are used in the above references, when the gray difference of the image to be segmented is small, there is large overlap within the gray value range, or the noise is serious, the accuracy of lesion area segmentation will be greatly reduced, especially when there is fat area near the breast nodule or the contrast is low, which will seriously affect the segmentation effect. Therefore, after initializing the nodule contour, Liu et al. [9] achieved accurate segmentation of breast nodules by improving the Chan-Vese model. Jiang et al. [10] further screened the detected lesion area set by using Support Vector Machine (SVM) on the basis of the original framework and refined and segmented the lesion area by Random Walk algorithm.

In recent years, ultrasound image segmentation algorithm based on neural network has been proposed continuously. For example, Li et al. [11] used encoder-decoder structure to segment amniotic fluid and fetal tissue in fetal ultrasound image effectively [11]. Zhang et al. [12] segmented the lesion area of lymph node ultrasound image from coarse to fine by improving Fully Convolutional Networks (FCN) [12]. Wu et al. [13] used Convolutional Neural Networks (CNN) to find the ROI in the fetal abdomen in the ultrasound image and evaluated the image quality by judging the clarity of description of the key structures of gastric vesicle and umbilical vein through another CNN, so as to screen high-quality ultrasound images for the measurement of fetal Abdominal Circumference (AC) [13]. Ma et al. [14] and Ma et al. [15] randomly cut the thyroid ultrasound image into multiple overlapping subgraphs, took the pixel ratio of normal tissue to lesion area as the classification label of each subgraph, trained the Deep Convolutional Neural Networks (DCNN), and finally used the trained DCNN for subgraph-level classification to realize the lesion area segmentation of thyroid ultrasound image. Among them, some scholars have achieved good research results in the field of breast ultrasound image nodule segmentation. Wang and Jiao [16] proposed an improved Simplified Pulse Coupled Neural Network (SPCNN) combined with fuzzy mutual information, which took the maximum fuzzy mutual information as the optimal decision criterion to obtain the result of whether each pixel belongs to the nodule area, and carried out morphological processing on the binary image after pixel classification to extract the breast lesion area [16]. Cao et al. [17] conducted multiscale transformation of breast ultrasound dataset and input it into the current mainstream target detection framework (including Fast R–CNN, Faster R–CNN, YOLO, and SSD) and backbone network (ZF-Net, VGG-16) [17]. The results show that SSD300 is more suitable for breast ultrasound lesion extraction. Although the deep learning method has made good achievements in the field of natural image processing, due to the characteristics of ultrasound imaging, breast ultrasound images have defects such as low resolution, low contrast, and a large amount of speckle noise and artifacts, and due to data sensitivity, there is a lack of large public breast ultrasound dataset. For the above reasons, the dataset-driven deep learning technology is disturbed in breast ultrasound image processing [18].

Vakanski et al. [19] integrated visual saliency into the U-Net segmentation model, drove the model to give priority to the highly significant spatial region features in the image, and obtained good experimental results on a small sample dataset composed of 510 breast ultrasound images [19]. Zhang et al. [20] proposed to optimize the convolution network AlexNet network with full connection structure to obtain the information features of the image, so as to complete the ultrasound image analysis of breast mass [20]. Ilesanmi et al. [21] optimized the processing of breast ultrasound images based on cascaded convolution network to realize image information recognition and segmentation [21]. Tang et al. [22] introduced the Transform Modal Ensemble Learning module into the nonlocal network of feature pyramid to construct the breast ultrasound image-aided diagnosis model and realize autonomous image analysis [22]. Fang et al. [23] constructed a full convolution network model M-Net to realize breast abnormal image analysis. The above methods still have limitations [23]. Due to deep network structure, the gradient of image segmentation model may disappear when recognizing images, resulting in the decline of image segmentation accuracy. At the same time, due to the large noise interference in the ultrasound image, there is independent phase interference when the depth model obtains the image features, resulting in the further decline of segmentation accuracy. This paper proposes a recognition method for breast tumor ultrasound images, improves the U-Net network, and integrates it with the hybrid attention mechanism network, which overcomes the problems of the current depth network model.

## 3. The Proposed Image Segmentation Method

**Figure 1:**
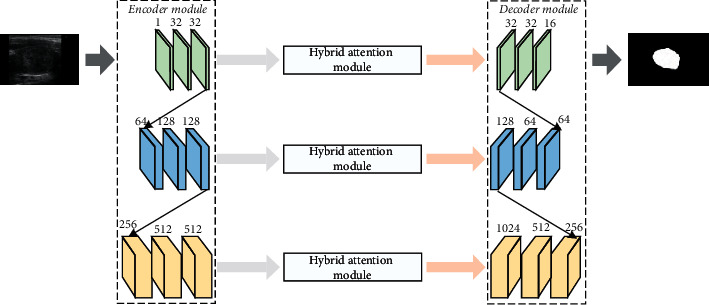
Image segmentation model based on improved residual U-Net network.

The overall framework of the proposed model is shown in Figure 1. The model takes U-Net as the backbone network structure and includes three parts: encoder module, hybrid attention module, and decoder module. In each level of the codec in Figure 1, the characteristic tensor passes through a residual block and a multiscale convolution block in turn. First, the image is subjected to residual multiscale convolution and downsampling encoding. At the same time, the feature map output at each level is copied to the decoder at the corresponding level through the attention module, and then the feature map obtained by downsampling is concatenate and upsampled. Finally, the breast tumor ultrasound image segmentation is realized through the loss function [24, 25]. In this method, the features extracted by the shallow encoder are connected to the corresponding level of the decoder through the attention module, which can give higher weight to the edge details in those shallow features and suppress the useless information or noise in the shallow features to a certain extent while retaining the edge information.

### 3.1. Residual Block

In order to extract more detailed information from the image and alleviate the problem of undersegmentation in low contrast areas and weak edges, residual blocks are introduced into U-Net to replace the traditional convolution layer. The use of residual blocks can solve the problem of gradient disappearance with the deepening of network layers. The calculation formula of residual block foundation is

(1) $$\begin{array}{c} u=c\left(v,w_{h}\right)+v, \end{array}$$

where *v* and *u* are, respectively, the input and output of the network, *w*_*h*_ is the parameter of *h*-th layer, and *c*(*v*, *w*_*h*_) is the residual mapping. Using residual block learning to fit a residual map *u* − *v* is easier than directly learning to fit an approximate identity map *u*. The residual block solves the problem of gradient disappearance in deep network. The partial derivative of formula (1) is

(2) $$\begin{array}{c} \frac{\partial u}{\partial v}=1+\frac{\partial c\left(v,w_{h}\right)}{\partial v}. \end{array}$$

Since the gradient of formula (2) is always greater than 1, the gradient will not disappear with the deepening of the number of network layers. In general, the feature dimension of *c*(*v*, *w*_*h*_) is different from that of *v*, so linear transformation parameter *w* is introduced to complete dimension matching. The calculation formula is

(3) $$\begin{array}{c} u=c\left(v,w_{h}\right)+w_{v}. \end{array}$$

**Figure 2:**
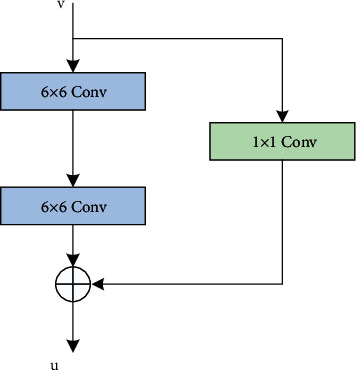
U-Net residual block structure.

The network structure of the residual block is shown in Figure 2. The input features are convoluted through two-standard 6 × 6 convolution layers to obtain the residual mapping, then the feature dimension matching is completed through the 1 × 1 convolution layer, and finally the feature fusion is completed through the Add operation. This module avoids the degradation of model performance caused by the disappearance of gradient, reduces the training difficulty of deep network, and only increases the 1 × 1 convolution layer, which will not increase the computational complexity of the model. At the same time, the module shortens the distance between the front and back layers, effectively improves the learning ability of features, and helps to extract more detailed information, which reduces the interference of low contrast between cells and background to the model to a certain extent.

### 3.2. Attention Module

**Figure 3:**
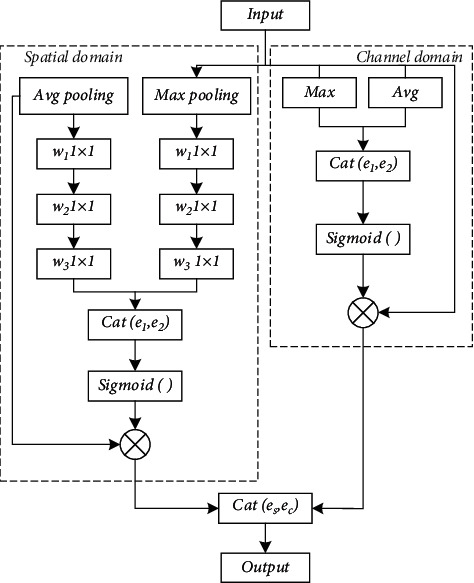
Space and channel attention module.

Although the spatial attention can find the relationship between features and task objectives from a global perspective and strengthen the attention to the target task, the spatial attention ignores the information in the channel and treats the feature map in each channel equally. Based on the attention of the channel, the global context information is extracted through the global maximum pooling and average pooling. As a guide, the correlation of the global information is judged from the perspective of channel correlation, and then the global features with high correlation are selected [26]. However, the attention of the channel is to pool the information in the channel globally, which ignores the local information in each channel. Combining the above two kinds of attention, a Spatial Channel Attention Block (SCAB) is proposed to allocate the weights of channel and spatial attention. The specific implementation process is shown in Figure 3.

As can be seen from the figure, the output *d* can be obtained by the following formula:

(4) $$\begin{array}{c} d=\text{Cat}\left[e_{s},e_{c}\right], \end{array}$$

where *e*_*s*_ is the feature with spatial attention and *e*_*c*_ is the feature with channel attention.

SCAB combines channel attention and spatial attention to obtain a comprehensive attention information. It not only analyzes the relationship between each pixel and the task target but also distinguishes the correlation between each channel and the task. Specifically, by inputting the input features *b* ∈ *R*(*G* × *P* × *Q*) into the spatial attention block and the channel attention block, respectively, the output of the spatial attention block *e*_*s*_ and the output of the channel attention block *e*_*c*_ can be obtained. *e*_*s*_ and *e*_*c*_ are combined to get the final output *d* ∈ *R*(*G* × *P* × *Q*).

The structure of feature extraction block in U-Net is two 2 × 2 convolutions to extract the input features. The feature learning is carried out by fusing the input with the output in the way of skip connection. This method has the following advantages: (1) it simplifies the learning process and enhances the propagation of gradient. Due to the inclusion of residual structural identity, the backpropagation can be carried out effectively. (2) This design can break the asymmetry of the network. If only a small number of hidden units in each layer of the network change their activation value for different inputs, and most hidden units respond the same way to different inputs, the problem of network degradation will occur. (3) The generalization ability of the network is enhanced. Residual SCAB (RSCAB) is a new feature extraction module combined with attention mechanism and residual structure.

The specific implementation of RSCAB is as follows:

(5) $$\begin{array}{c} d_{\text{att}}=\text{SCAB}\left(\alpha \;d+\beta \right), \end{array}$$

(6) $$\begin{array}{c} b=\alpha \text{Cat}\left[d_{\text{att}},d\right]+\beta , \end{array}$$

where the function of 1 × 1 convolution is to raise the input dimension. *d*_att_ is the feature map obtained by raising dimension and SCAB, and *b* is obtained by feature extraction again after feature fusion of *d*_att_ and *d*.

By embedding the attention transmitted by the residual connection, the module enables the network to obtain the location of the task target in the feature extraction stage. It has the following advantages:

(l) The shallow attention features are transferred to the deep by residual connection, which can pay more attention to the feature learning of the task target area.
(2) In this way, the interaction between layers can be closer, so as to facilitate the calculation of feature information.
(3) It enhances the information and gradient transmission between networks and facilitates the training of deeper networks.

### 3.3. Loss Function

In the classical U-Net model training, the output result of Softmax is used to calculate the cross entropy, which is regarded as the optimization function of the whole network. In medical image segmentation, there are usually only two classifications: lesions and background. At this time, the loss function is Binary Cross Entropy (BCE), and its formula is

(7) $$\begin{array}{c} {\text{loss}}_{\text{BCE}}=-\frac{1}{N}\left[t_{j}\sum_{j=1}p_{j}+\left(1-t_{j}\right)\sum_{j=1}\left(1-p_{j}\right)\right], \end{array}$$

where *N* is the total number of pixels of the input image, *t*_*j*_ is the truth label of the *j*-th pixel, *t*_*j*_ ∈ [0,1] is the probability that the corresponding pixel is predicted as the foreground, 0 represents the background, and 1 represents the mass. When the number of foreground pixels is far less than the number of background pixels, that is, the number of *t*_*j*_=0 is far greater than the number of *t*_*j*_=1, the component *t*_*j*_=0 in the optimization process will dominate, making the model seriously biased towards the background.

There is a serious imbalance between foreground and background pixels in the dataset. Using the binary classification cross entropy loss function alone may mislead the optimization direction of the model and finally can only predict the meaningless background area with a larger area.

Dice similarity coefficient is a set similarity measurement function, which is usually used to calculate the similarity of two types of pixel samples, with a value range of [0,1]. When the number difference between the two types of samples is huge, loss_DICE_ can alleviate the problem of sample imbalance to a certain extent. loss_DICE_ is defined as

(8) $$\begin{array}{c} {\text{loss}}_{\text{DICE}}=1-\frac{2\sum_{j}p_{j}t_{j}+\gamma }{\sum_{j}p_{j}^{2}+\sum_{j}t_{j}^{2}+\gamma }, \end{array}$$

where *γ* is a constant added to maintain numerical stability and prevent denominator from changing to 0. In breast image segmentation, due to the extreme imbalance between tumor pixels and background pixels, using Dice loss function alone will make the whole optimization process unstable and reduce the reliability of the index. Therefore, in the training process, two loss functions are used to constrain the network training, and a weighted composite loss function loss_NEW_ is proposed, which is defined as follows:

(9) $$\begin{array}{c} {\text{loss}}_{\text{NEW}}=\tau {\text{loss}}_{\text{BCE}}+{\text{loss}}_{\text{DICE}}, \end{array}$$

where *τ* is a constant weight to control the balance of optimization strength between cross entropy loss and Dice loss. After testing different *τ* values, it is found that better segmentation effect can be achieved at *τ*=1.5 × 10^−3^.

### 3.4. Network Training

Deep learning network training aims at minimizing the loss function and iteratively updating the weight parameters of the model. It consists of two parts: weight initialization and updating algorithm.

#### 3.4.1. Weight Initialization

The weight initialization method in deep learning network model is very important, which affects the convergence performance and convergence speed of network model training. With the deepening of layers of neural network, the optimization method based on gradient descent is prone to gradient explosion and gradient disappearance, and a good weight initialization method can alleviate this problem.

The ReLU function is used as the activation function. The specific operation is to initialize the weight parameters of the convolution kernel using a Gaussian distribution with a mean value of 0 and a standard deviation of $\sqrt{2/\delta }$, where *δ*=*k*_*r*_^2^*z*_*r*−1_, in which *k*_*r*_ refers to the size of the convolution kernel of *r* convolution layer and *z*_*r*−1_ represents the number of convolution kernels in the *r* − 1 layer.

#### 3.4.2. Weight Update Method

The training of neural network is a process of finding the optimal value, and this process is actually a process of iteratively updating the weights. The optimization algorithm contains different weight updating methods. The optimization algorithm used in this paper is Adam algorithm. Adam algorithm is a stochastic optimization method of adaptive momentum, which iteratively updates the training parameters by combining the first-order moment and second-order moment information of the gradient. The specific update calculation is as follows:

(10) $$\begin{array}{c} y_{t}\leftarrow \nabla_{\theta }x_{t}\left(\theta_{t-1}\right), \end{array}$$

(11) $$\begin{array}{c} o_{t}\leftarrow \varphi_{1}\cdot o_{t-1}+\left(1-\varphi_{1}\right)\cdot y_{t}, \end{array}$$

(12) $$\begin{array}{c} z_{t}\leftarrow \varphi_{2}\cdot z_{t-1}+\left(1-\varphi_{2}\right)\cdot y_{t}^{2}. \end{array}$$

(13) $$\begin{array}{c} {\hat{o}}_{t}\leftarrow \frac{o_{t}}{\varphi_{1}}, \end{array}$$

(14) $$\begin{array}{c} {\hat{z}}_{t}\leftarrow \frac{z_{t}}{\varphi_{2}}, \end{array}$$

(15) $$\begin{array}{c} \theta_{t}\leftarrow \theta_{t-1}-\frac{\varepsilon \cdot {\hat{o}}_{t}}{\left(\sqrt{{\hat{z}}_{t}+\sigma }\right)}, \end{array}$$

where *t* is the number of iterations, *x*(*θ*) refers to the loss function, *θ* is the training parameter, that is, the weight or bias, *y*_*t*_ is the gradient calculated by the derivation of the loss function from the training parameter, *φ*_1_ is the first-order moment attenuation coefficient, *φ*_2_ is the second-order moment attenuation coefficient, *ε* is the learning rate, *o*_*t*_ is the first-order moment of the gradient *y*_*t*_, *z*_*t*_ is the second-order moment of the gradient *y*_*t*_, ${\hat{o}}_{t}$ and ${\hat{z}}_{t}$ are the deviation correction of *o*_*t*_ and *z*_*t*_, and *σ* is a positive number to prevent the denominator from being zero.

The value of each parameter is as follows: *φ*_1_=0.95, *φ*_2_=1.00, *ε*=0.00015, and *σ*=1.5 × 10^−8^. Adam algorithm combines the characteristics of momentum method and RMSprop method to jump out of the local optimal solution as much as possible and accelerate the convergence speed of the network.

## 4. Experiment and Analysis

The computer hardware configuration of the experimental simulation is Intel Core i5-10400 and Nvidia GeForce RTX 3080Ti. The network model is trained and tested using the Keras open-source library with TensorFlow as the backend.

### 4.1. Experimental Dataset and Preprocessing

The experimental dataset is the Wisconsin Diagnostic Breast Cancer (WDBC) dataset. Breast ultrasound image has three inherent characteristics, including high noise, low contrast, and nonuniformity. Therefore, the segmentation of breast ultrasound image is still a challenging task. At the same time, a series of preprocessing operations are needed to process this kind of image.

#### 4.1.1. Cutting of Region of Interest

For breast ultrasound image segmentation task, the region of interest is breast tumor. In the original breast ultrasound image, the proportion of tumor area is often small. In order to improve the efficiency of subsequent superpixel generation, the region of interest of the original image is cut. In the original breast ultrasound image, the operator determines two diagonal points and then draws a rectangular box. The box needs to meet two requirements: (1) the rectangular box contains breast tumors and (2) the area of the tumor accounts for about 30% of the area of the rectangular box. The region of interest is cut from the original Image, which greatly reduces the interference of irrelevant regions and improves the efficiency of the algorithm.

#### 4.1.2. Image Denoising

Breast ultrasound image is full of noise, and the main noise is speckle noise, which needs denoising. In this paper, bilateral filter is used. The ultrasound image is denoised by bilateral filter. The filtering process integrates the gray similarity and spatial information between pixels, which can not only preserve the tumor edge but also reduce the noise.

#### 4.1.3. Contrast Enhancement

**Figure 4:**
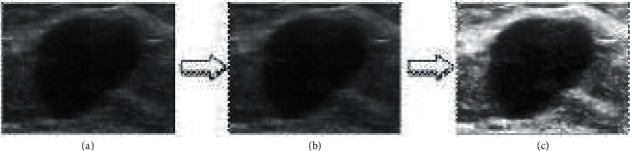
Process of image preprocessing. (a) Captured image. (b) Denoised image. (c) Enhanced image.

In addition to high noise, breast ultrasound images also have the characteristics of low contrast. Specifically, the gray values of breast tumor area and some adjacent normal tissue areas are close, and the difference is small. It is easy to mistake this part of normal tissue area for tumor area. Therefore, it is necessary to enhance the contrast and the method of gray histogram equalization is adopted. The mathematical description of the specific transformation of histogram equalization is as follows:

(16) $$\begin{array}{c} e_{j}=U\left(v_{j}\right)=\sum_{i=1}^{j}G\left(v_{i}\right)=\sum_{i=1}^{j}\frac{c_{i}}{c},\;j=1,2,\ldots ,J, \end{array}$$

where the number of pixels of the input image is *c*, *J* represents the gray level, *G*(*v*_*i*_), *i*=1,2,…, *J* represents the gray histogram of the region of interest, *c*_*i*_ is the number of pixels with gray level *i*, *v*_*i*_ is the gray value of the image before processing, and *e*_*j*_ is the gray value after equalization. The flow of image preprocessing is shown in Figure 4.

### 4.2. Evaluation Index

In addition to the loss function mentioned above, the ultrasound image segmentation reference indices with universality, objectivity, and quantifiability are still selected to evaluate the above algorithm, specifically including Dice, Intersection over Union (IoU), Hausdorff Distance (HD), and Mean Absolute Deviation (MAD). Dice is a regional error evaluation method. The pixels of the segmented region and the actual lesion area are calculated in a certain way. The higher the value, the better the segmentation effect. The principle of IoU is similar to the previous index, which refers to the overlap rate between the segmented area and ROI. In the ideal state, the ratio is 1; that is, the two completely overlap. Hausdorff Distance (HD) measures the maximum distance between the boundary generated by segmentation and the true subset of the actual lesion area. The lower the value, the smaller the boundary error. Mean Absolute Deviation (MAD) reflects the average dispersion between the real lesion area and the segmented area pixels. The lower the value, the more accurate the segmentation. The calculation formulas are shown in (17)–(20).

(17) $$\begin{array}{c} \text{Dice}=\frac{2\left|R\cap G\right|}{\left(\left|R\right|+\left|G\right|\right)}, \end{array}$$

where *R* is the segmentation result area and *G* is the real lesion area.

(18) $$\begin{array}{c} \text{IoU}=\frac{\left|R\cap G\right|}{\left|R\cup G\right|}, \end{array}$$

(19) $$\begin{array}{c} \text{HD}=\max\left\{\underset{x\in R}{\max}\left\{d\left(x,G\right)\right\},\underset{y\in G}{\max}\left\{d\left(y,R\right)\right\}\right\}, \end{array}$$

where *d*(*x*, *C*)=max_*y*∈*G*_{‖*x* − *y*‖}, *C*=*R* or *G*.

(20) $$\begin{array}{c} \text{MAD}=\frac{1}{2}\left(\sum_{x\in R}\frac{d\left(x,G\right)}{N_{R}}+\sum_{y\in G}\frac{d\left(y,R\right)}{N_{G}}\right), \end{array}$$

where *N*_*R*_ is the total number of pixels in the segmentation result area and *N*_*G*_ is the total number of pixels in the real lesion area.

### 4.3. Ultrasound Image Segmentation Model Analysis

**Figure 5:**
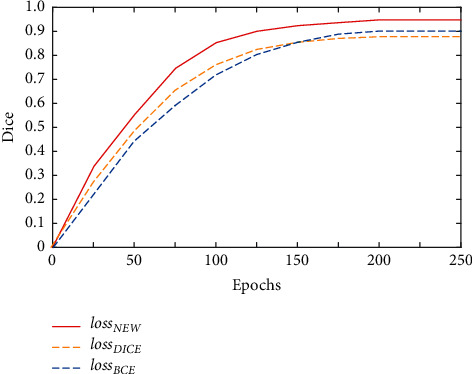
Changes of Dice value under different loss functions.

In order to prove the feasibility of the proposed model, first, the performance of the segmentation model is evaluated.  Figure 5 shows the change of Dice value curve on the validation set when the proposed network uses different loss functions in the training process, including loss function loss_DICE_, loss function loss_BCE_, and composite weighted loss function loss_NEW_ proposed in this experiment.

It can be seen from Figure 5 that when using the composite weighted loss function loss_NEW_ for training, the Dice value curve rises steadily, and the segmentation accuracy can reach more than 0.95 on the validation set in the later stage of training, while the other two loss functions enter the training bottleneck after reaching more than 0.85, and the Dice values do not break through 0.9 in the end.

**Figure 6:**
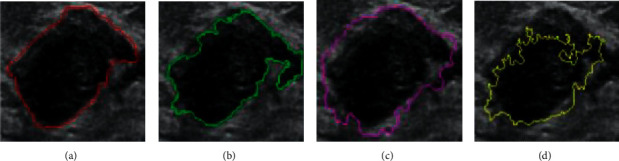
Image segmentation results under different methods. (a) Manual label. (b) Proposed model. (c) Reference [20]. (d) Reference [23].

Using [20, 23] as comparison methods, the experimental datasets are analyzed in the same environment. As shown in Figure 6, different colors represent the segmentation contour obtained by different methods. Among them, (a) is the image with the contour manually labeled by the doctor, (b) is the result of the segmentation model proposed in this paper, (c) is the segmentation result of the method in [20], and (d) is the segmentation result of the method in [23].

According to the qualitative experimental results shown in Figure 6, the segmentation performance of the segmentation model proposed in this paper is better than the comparison algorithms. Although there are still some defects in the extracted edge, compared with the other two methods, it is the closest to the manually labeled edge and has better visual effect. Methods of [20, 23] produce a certain degree of undersegmentation, the edge obtained by [23] is also rough, and [20] produces obvious oversegmentation. The reason is that the comparison method cannot obtain the data features in the sample set well and ignores some local features in the image. The segmentation model proposed in this paper adds the fusion attention mechanism module to the model, which can realize the global data feature extraction and analysis and help to realize the accurate image segmentation combined with context-related information. In addition, due to the improvement and optimization of the loss function, it can also make the model perform calculation and analysis in the direction of global optimization, so as to avoid the interference of noise on the recognition results.

**Figure 7:**
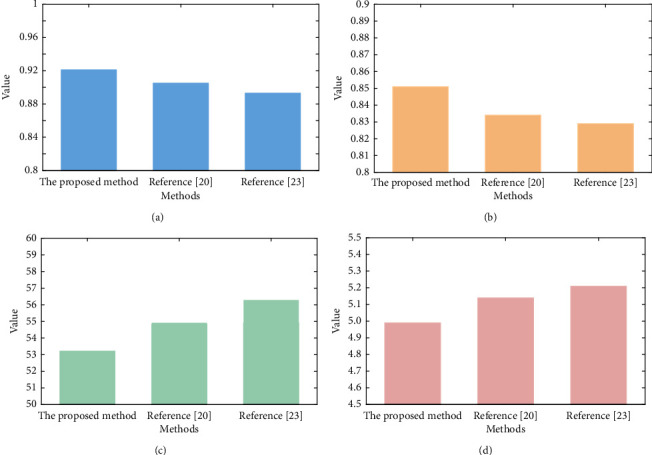
Comparison of segmentation results of different methods. (a) Dice index. (b) IoU index. (c) HD index. (d) MAD index.

Further quantitative analysis is carried out.  Figure 7 shows the comparison of quantitative segmentation results of different methods.

It can also be seen from Figure 7 that, for the analysis of ultrasound images of complex breast tumors, the segmentation errors of the comparison methods are large, and the accuracy of the proposed model is higher than those of other comparison methods. The Dice value of the proposed method is 0.921, which is 0.016 and 0.028 higher than those in [20, 23]. In terms of nodule edge segmentation accuracy, the HD index of the proposed model is 3.08 lower than that in [23]. IoU is 0.851 and MAD is 4.99, which are better than the evaluation indices in [20, 23]. This shows that the proposed model can effectively improve the accuracy of ultrasound image region segmentation.

## 5. Conclusion

A breast tumor ultrasound image segmentation method based on improved residual U-Net network is proposed. The U-Net network model is improved by introducing residual block optimization to avoid the disappearance of gradient caused by too deep network structure. Furthermore, the hybrid attention mechanism network is introduced into the ultrasound image segmentation model to obtain the global information features of the sample dataset and effectively improve the image analysis performance of the analysis model. The experimental results show that the proposed method can realize the analysis and processing of actual complex breast tumor ultrasound images and provide reliable medical diagnosis assistance for medical staff.

This paper mainly studies the shape feature constraints of breast nodules, but the situation of breast ultrasound images is complex and changeable. In real life, there are other types of difficult samples based on texture characteristics such as artifact and calcification. How to automatically mine these different types of difficult samples becomes the next research work.

## Data Availability

The data used to support the findings of this study are included within the article.
